# Minnesota Medical Practice Act

**Published:** 1884-11

**Authors:** 


### Minnesota Medical Practice Act.

The Supreme Court of the State of Minnesota recently decided that the Medical Practice Act is constitutional, and that the Board of Medical Examiners has power to refuse a license to practice to any person on the ground of unprofessional conduct or incompenency. It also has the power to revoke any license already granted, provided the holder is guilty of unprofessional or felonious conduct.

This decision of the Supreme Court of Minnesota sustains and strengthens the recent decisions of the Supreme Court of Illinois. If the good work goes on there will soon be no resting place for medical quacks and incompetents.

Now that a decision has been had, the Board intends taking more vigorous steps and there will, probably, be heard a "squawk" all along the line of human vampires.—*Northwestern Lancet.*

